# Cardiovascular disease and arsenic exposure in Inner Mongolia, China: a case control study

**DOI:** 10.1186/s12940-015-0022-y

**Published:** 2015-04-12

**Authors:** Timothy J Wade, Yajuan Xia, Judy Mumford, Kegong Wu, X Chris Le, Elizabeth Sams, William E Sanders

**Affiliations:** United States Environmental Protection Agency, Office of Research and Development, Chapel Hill, North Carolina USA; Inner Mongolia Centers for Endemic Disease Control and Research, Huhhot, Inner Mongolia China; University of Alberta, Edmonton, Alberta Canada; University of North Carolina, Chapel Hill, North Carolina USA

**Keywords:** Arsenic, Cardiovascular disease, China, Drinking water

## Abstract

**Background:**

Millions of people are at risk from the adverse effects of arsenic exposure through drinking water. Increasingly, non-cancer effects such as cardiovascular disease have been associated with drinking water arsenic exposures. However, most studies have been conducted in highly exposed populations and lacked individual measurements.

**Objective:**

To evaluate the association between cardiovascular disease and well-water arsenic exposure.

**Methods:**

We conducted a hospital based case control study in Inner Mongolia, China. Cases and controls were prospectively identified and enrolled from a large hospital in the Hangjin Hou area. Cases were patients diagnosed with cardiovascular disease and controls were patients free from cardiovascular disease, admitted for conditions unrelated to arsenic exposure. Water from the primary water source and toenail samples were collected from each subject and tested for inorganic arsenic.

**Results:**

Arsenic exposures were moderate with mean and median arsenic exposures of 8.9 μg/L and 13.1 μg/L, respectively. A total of 298 cases and 275 controls were enrolled. The adjusted odds ratio (AOR) and corresponding 95% confidence interval (95% CI) for a 10 μg/L increase in water arsenic were 1.19 (95% CI: 1.03, 1.38). Compared to exposures less than 10 μg/L, the AOR for water arsenic exposures above 40 μg/L was 4.05 (95% CI: 1.1-14.99, p = 0.04). Nail arsenic above 1.38 μg/g was also associated with an increased risk of cardiovascular disease.

**Conclusions:**

By using standardized case definitions and collecting individual measurements of arsenic, this study addressed several limitations of previous studies. The results provide further evidence of the association between cardiovascular disease and arsenic at moderate exposures.

**Electronic supplementary material:**

The online version of this article (doi:10.1186/s12940-015-0022-y) contains supplementary material, which is available to authorized users.

## Background

Worldwide, millions of people are at risk from the adverse effects of arsenic exposure through drinking water. Studies in Taiwan, Argentina and Chile have documented increased bladder, lung and skin cancer mortality as a result of arsenic exposure [1-3]. More recently, exposure to waterborne arsenic has been associated with non-cancer health effects such as cardiovascular disease [4-8] and neurological disorders [9-12].

Epidemiological studies have associated arsenic exposures with cardiovascular and peripheral vascular health effects including Blackfoot disease, hypertension, atherosclerosis, ischemic heart disease, vascular disease mortality, and abnormal electrocardiograms (ECG), including prolonged QT-interval [13-16]. Acute arsenic toxicity is known to cause cardiovascular effects including abnormal ECGs [17] and ventricular fibrillation [18,19]. Arsenic trioxide treatment for promyelocytic leukemia is also known to cause prolonged QT intervals, a risk factor sudden cardiac death, by blocking repolarizing potassium currents [20,21]. Arsenic is believed to induce these cardiovascular effects through several potential mechanisms including inflammation in vascular tissues, oxidative stress, endothelial injury and smooth muscle cell proliferation [17].

Increased cardiovascular mortality was observed in populations in Taiwan exposed to extremely high levels of arsenic in well water [6,13,16,22,23]. Most of these studies, however, were retrospective, lacked individual measures of arsenic or cardiovascular disease (CVD), and were conducted in populations with high arsenic exposures (e.g., >600 μg/L). There is increasing evidence that the cardiovascular effects of arsenic may manifest at considerably lower arsenic exposures. Two recent studies in the United States observed associations with cardiovascular disease (CVD) and coronary heart disease in populations exposed to only moderate levels of arsenic (<100 μg/L) [24,25]. A recent review of the topic concluded that at “low-moderate arsenic levels, high quality prospective studies including individual-level exposure assessment and standardized CVD outcomes are needed to understand the role of arsenic as a CVD risk factor” [26].

In order to better understand the association between waterborne arsenic and cardiovascular disease, especially at low to moderate levels of exposure, we conducted a hospital-based case control study in Inner Mongolia, China in a population exposed to arsenic contaminated well water.

## Methods

### Study location

**Figure 1:**
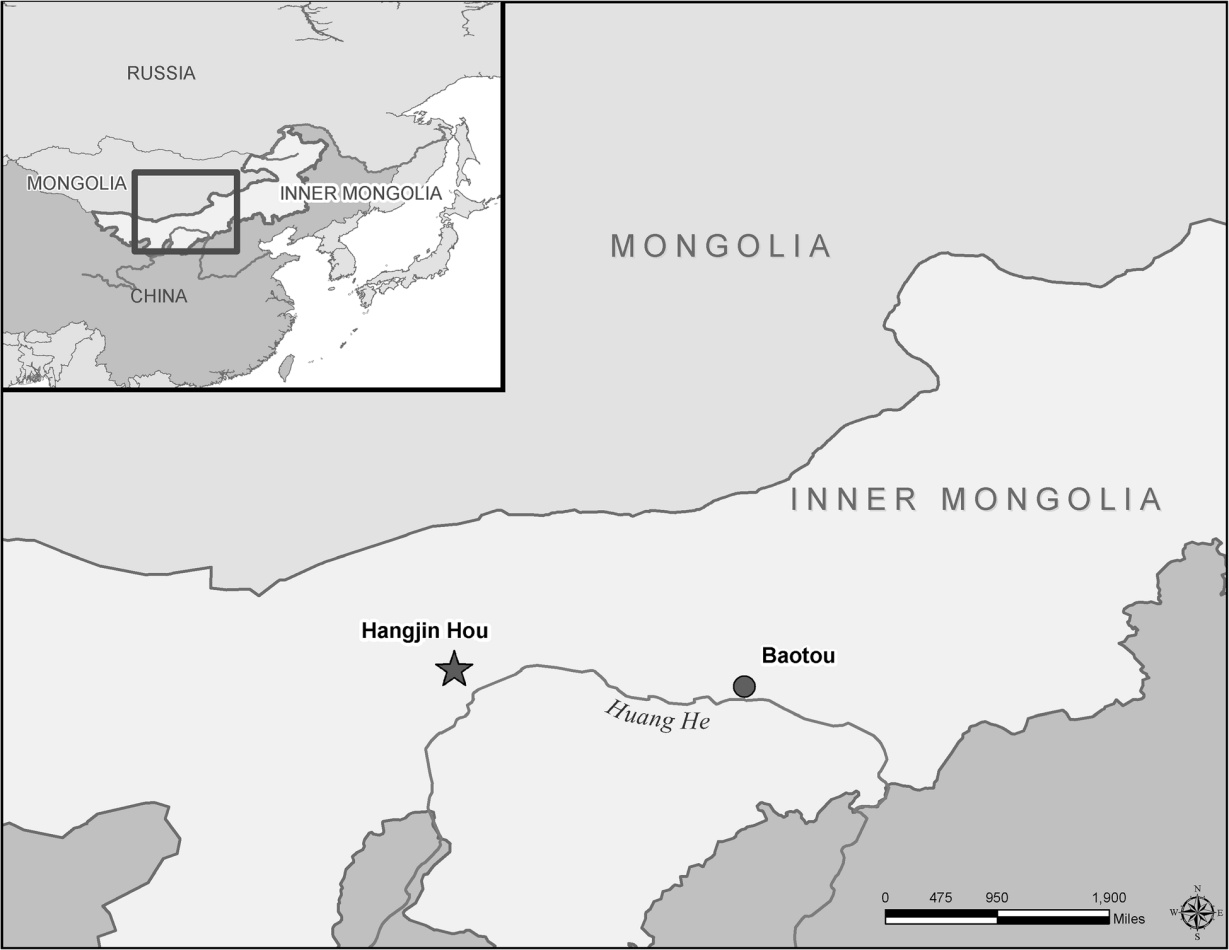
Study site.

The study was conducted in the Bayingnormen (Ba Men) region of Inner Mongolia, China. The Ba Men region is located on the Hetao Plain, north of the Huang He (Yellow) River in western Inner Mongolia, China (Figure 1). In the late 1970s, the primary source of drinking water shifted away from large shallow wells to deeper artesian wells due to water scarcity and contamination of the shallow wells. These artesian wells were often contaminated by naturally occurring arsenic. Characteristic arsenic-induced skin lesions were reported in the region in the early 1990s. Several studies have documented the prevalence of adverse health effects associated with arsenic exposure in this region [4,27-29].

Ba Men is a rich agricultural region and most residents of the region are farmers. Ma et al. reported that drinking water was the only significant source of arsenic exposure and that arsenic containing pesticides had not been used in the region [27]. Samples from soil, certain foods (wheat, corn, sunflower seeds, beans, melon and vegetables), and surface water were found to have arsenic levels lower than China national standards [30]. Rice, another potentially important source of inorganic arsenic exposure [31], is a less common staple food in this part of China than locally produced foods made with wheat grain. According to our collaborators from the region, rice is not grown locally. The residents occasionally consume freshwater fish from the Huang He River, and only very rarely seafood from the ocean [32].

### Study site and population

We recruited cases with cardiovascular disease and controls from a regional hospital in the Hangjin Hou (Hang-Hou) area of Ba Men, Inner Mongolia (Figure 1).

Hang-Hou hospital serves a population base of approximately 300,000 from a region of approximately 1800 km^2^. Hang-Hou hospital is equipped with modern facilities, including equipment necessary (echocardiogram and interventional laboratories) for diagnosing cardiovascular disease. Cardiologists at the hospital reported a minimum of 900 patients were admitted annually for cardiovascular disease.

### Enrollment and informed consent

Local medical staff trained on the study procedures, protocols and consent processes described the study and offered enrollment to newly admitted patients with chest pain or suspected cardiovascular disease, as well as to suitable potential controls. Patients interested in participating were provided details about the study and read and signed an informed consent form. Study materials and procedures were approved by the Institutional Review Board for the University of North Carolina at Chapel Hill (UNC). All study staff were trained on the informed consent process and were provided training on the ethical treatment of human research subjects. Ethical certification, approval and permission to conduct the study were also obtained from the Inner Mongolia Centers for Endemic Disease Control and Research (IMCDC) and from the Director of Hang-Hou hospital.

Following enrollment, clinical information and tests were obtained if not available. Study staff administered a standardized questionnaire to acquire information regarding personal and family health history, diet, other health habits and behaviors, occupation and residence histories. Toenails and/or fingernails were collected while the patient was in hospital or at the subsequent home visit. Appointments were scheduled to visit the patient home to collect water samples. All home visits were conducted within one month of enrollment at the hospital.

### Case definition and selection

Inclusion criteria for cases were: 1) Between 18 and 70 years of age; 2) Met one of the three case definitions (described below) 3) Resident of Hang-Hou county. Potential cases were excluded if they had severe valvular disease (e.g., aortic stenosis).

Cases were defined as patients meeting any one of the three following conditions:

1. Acute Myocardial Infarction (MI). Defined as having 2 of 3 findings:
  Recent chest pain consistent with angina for approximately 30 minutes or more.; ECG changes consistent with MI: ST segment elevation greater than or equal to 1.0 mV in 2 contiguous leads (2 leads following each other); Positive enzymes: Elevation of CK/MB or troponin consistent with MI.
2. Cardiomyopathy. Defined ejection fraction less than or equal to 35% without valvular heart disease.
3. Chest pain suggestive of angina with any one of the following: changes on ECG consistent with previous MI (Q-waves); documented enzyme changes consistent with previous MI; changes in serial ECG tracings (for example T-wave changes); ECG changes on treadmill stress (exercise) testing consistent with injury or ischemia.
  The following criteria were used to determine an abnormal treadmill stress test: ST segment elevation greater than 1.0 mV in contiguous leads that do not normally have Q waves (other than V1 or a VR); ST depression greater than or equal to 2.0 mm which is horizontal or downsloping measured ST segment greater than or equal to 0.08 seconds after QRS termination.

At the discretion of the attending physician, borderline cases (i.e., cases with chest pain suggestive of angina but no evidence of previous MI from ECG changes or enzymes) were asked to complete a treadmill stress test utilizing a standard Bruce protocol [33]. ECG tracings from the stress test were used to confirm if the patient had ischemia consistent with cardiovascular disease. Case reports, which documented the physician’s diagnosis and assessment, results of relevant laboratory tests, a copy of the ECG and echocardiogram, were translated to English by IMCDC staff and provided to the study cardiologist who reviewed and confirmed the cases. Cases which could not be confirmed to meet the criteria were excluded.

### Control selection

Controls were between 18 and 70 years of age and residents of Hang-Hou County. Controls were frequency matched to cases by sex and age (+/-5 years). Controls were patients visiting Hang-Hou hospital without cardiovascular disease as defined above and admitted for conditions not known to be associated with arsenic exposure. The exclusionary admission conditions included: skin, lung, gastrointestinal (GI) tract and bladder cancers, stroke, neuropathy, facial nerve palsy, diabetes, hypertension (systolic blood pressure > 140; diastolic blood pressure > 90), gangrene, amputation of limb or extremity (resulting from gangrene or other peripheral vascular insufficiency), and cirrhosis of the liver. Acceptable conditions for control selection included, for example: injury, childbirth, general exams, and infections. In addition to patients admitted to the hospital, we considered outpatient visitors as potential sources of controls, for example patients reporting for routine examinations, physicals or check-ups.

Screened subjects were excluded from qualifying as a control if any of following were present: ECG showed evidence of cardiovascular disease: existing heart disease; peripheral vascular disease; abnormal left ventricular ejection fraction on echocardiogram (<55%).

### Nail sample collection and arsenic measurement

Study subjects were asked to provide toenail samples under the supervision of study staff in hospital or at their place of residence using a clean nail clipper (provided by study staff). One to 3 mm of toenail was collected from each toe and pooled in a sealed plastic bag. When toenails could not be collected, or a relatively small amount of toenail was collected, fingernail clippings were collected. Approximately 30% of subjects provided nail samples at the subsequent home visit because their nails were too short at the time of their hospital stay. Of the subjects with missing nail samples, 14 were not present at the time of the home visit because they had moved (n = 4) or were deceased (n = 10). The remaining 26 either refused or did not have enough nail for collection and analysis. Toenails were stored and shipped to the United States Environmental Protection Agency (EPA) laboratory in North Carolina at ambient temperature for processing and aliquoting. The nail samples were cleaned as described previously [34] by sonification in HPLC-grade water to remove debris from the nail surface followed by acetone wash to dry and remove additional organic contaminants. Toenails were analyzed for arsenic using Instrumental Neutron Activation Analysis (INAA) at North Carolina State University Nuclear Services Department (Raleigh, NC, USA) [35]. Samples were tested in triplicate and the average result, detection limit and uncertainty was reported for each sample. The average limit of detection was 0.16 μg/g and the average uncertainty was 15%.

Fewer than 6% of nail samples were below the limit of detection (LOD) and these were assigned a value of $LOD/\sqrt{2}$. Nail samples had a variable limit of detection dependent on the mass of the sample. When possible, toenails were used for nail arsenic measurements. For 28 subjects of the 566 that provided nail samples (<5%), only fingernail samples were collected. Because in these samples, fingernail and toenail arsenic were highly correlated for those who provided both (r = 0.73), regression coefficients of a regression model of toenail arsenic with fingernail arsenic and age as independent variables were used to impute values for missing toenail arsenic samples (single imputation procedure) for the 28 subjects that provided fingernail but not toenail samples according to the following regression model: Toenail Arsenic (μg/g) = 0.988 + (0.8165 × Fingernail Arsenic (μg/g))-(0.0108 × Age (years)).

### Water sample collection and arsenic measurement

At the time of the home visit, study staff collected samples of water from the primary drinking water source. Subjects were assigned a water arsenic exposure based on their primary source of drinking water. Samples were collected in acid-washed tubes, transported to the University of Alberta on blue ice and analyzed for total arsenic using inductively coupled plasma mass spectrometry (ICPMS) as described previously [36]. For shared wells or subjects using distributed or municipal water, a single sample was collected and tested to represent arsenic exposures. The detection limit by ICPMS was 0.1 μg/L. The average coefficient of variation was 1.9%. Two water arsenic samples were measures below the LOD and were assigned a value of $LOD/\sqrt{2}$.

### Covariate measurement and assessment

In addition to age category and sex, the following covariates were considered in multivariate models as potential confounders of the association between arsenic and cardiovascular disease: diet, body mass index (BMI); occupation (unemployed, farming, industry, professional and other); education (None or some primary school; primary or some high school; completed high school); smoking (non-smoker, former smoker, or current smoker); alcohol use (yes or no); and family history of hypertension, diabetes or heart disease. With the exception of BMI, which was based on measured height and weight in the hospital, these were derived from responses to the questionnaire and interview.

To account for diet, subjects were asked how often in a month they usually eat various types of foods including fish, beef, grains, pork, beans, vegetables and fruit. For analysis, these responses were combined into summary estimates of servings per month for fruits and vegetables; protein other than meat (milk, soy) and meat (beef, chicken, pork, other meat) by assigning numerical values of 0; 0.5; 3; and 10 servings to the categorical responses never, less than 1, 1 to 5 and over 5, respectively. Because nearly all subjects reported eating grain (rice, wheat or corn) regularly, it was not included in additional analysis.

### Statistical analysis

Cardiovascular disease case status (present or absent) and arsenic measures (nail and water) were the outcome and exposures of interest. Tabulations of nail and water arsenic categories and case/control status were compared with demographic and health characteristics (smoking, age, sex, income, occupation, diet) to identify correlates of cardiovascular disease and water and nail arsenic.

Logistic regression models were used to assess the association between case status and arsenic exposures controlling for the variables described above. Models were fit with all the variables and a final model was selected by sequential backwards removal of each variable, maximizing the Akaike’s information criterion (AIC) [37]. Only alcohol use was excluded by the selection procedure, so all models included age, sex, diet, BMI, occupation, education, smoking, and family history of hypertension, diabetes or heart disease. Water arsenic and nail arsenic were modeled as continuous variables and also as categorical variables. Water arsenic was categorized as follows: less than 10; 10-40 and over 40 μg/L. These categories were based on the current EPA regulatory standard [38] and World Health Organization guideline value [39] of 10 μg/L for the lowest category and just below the previous EPA standard of 50 μg/L for the upper category. Because there was a limited range of exposure, additional categories were not considered. Nail arsenic was categorized based on percentiles of the distribution to contrast the lowest exposures and the highest exposures as follows: 0.11-0.28 μg/g (<10^th^ percentile); 0.29-1.37 μg/g (11-90^th^ percentile) and 1.38-34.2 μg/g (>90^th^ percentile).

### Study population and recruitment

Case and control recruitment began in July 2006 and ended in December 2011. In total 344 cases and 275 controls were recruited. Forty-five cases were excluded following review because they did not meet case criteria, leaving 298 cases. The general older age of cardiovascular disease patients and the younger age of most acceptable controls (injuries, pregnancy, infections), resulted in fewer controls than originally anticipated.

Of the 298 cardiovascular cases, 22 met the case definition of cardiomyopathy; 207 for acute MI; and 75 for previous MIs. Six of the cardiomyopathy cases also met criteria for a previous MI. The most common types of admissions for controls were upper respiratory symptoms (colds, flu, allergies), injuries, gastrointestinal symptoms, and regular examinations.

## Results

Characteristics of cases and controls are shown in Table 1. More cases reported smoking, having a family history of diabetes, hypertension or heart disease (Table 1), and eating beef, pork and animal fat (data not shown). Cases also had a higher average BMI (24.6 vs. 23.8) and 26% of cases had a BMI above 26 compared to 16% of controls. Cases were also slightly older than controls (mean age 57.4 vs. 54.8).

**Table 1:** **Characteristics of cases and controls** ^**a**^

	**Control (N = 275)**	**Case (N = 298)**	**Total (N = 573)**
	**N(%)**	**N(%)**	**N(%)**
*Sex*			
Male	185 (67%)	209 (70%)	394 (69%)
Female	90 (33%)	89 (30%)	179 (31%)
*Age*			
21-40	23 (8%)	21 (7%)	44 (8%)
41-50	59 (22%)	49 (16%)	108 (19%)
51-60	104 (38%)	91 (31%)	195 (34%)
61-70	89 (32%)	137 (46%)	226 (39%)
*Occupation*			
Unemployed	41 (15%)	19 (7%)	60 (11%)
Farming	116 (42%)	185 (64%)	301 (54%)
Industry	19 (7%)	23 (8%)	42 (8%)
Professional	49 (18%)	41 (14%)	90 (16%)
Other	50 (18%)	20 (7%)	70 (12%)
*Smoking*			
Never Smoker	134 (49%)	86 (30%)	220 (39%)
Former Smoker	25 (9%)	91 (31%)	116 (21%)
Current Smoker	115 (42%)	115 (39%)	230 (41%)
*Drinks Alcohol*			
No	164 (60%)	158 (54%)	322 (57%)
Yes	111 (40%)	135 (46%)	246 (43%)
*Family history of cardiovascular disease*			
No	227 (83%)	141 (48%)	368 (65%)
Yes	48 (18%)	152 (52%)	200 (35%)
*Education*			
None or some primary school	65 (24%)	97 (33%)	162 (29%)
Some high school	125 (46%)	139 (48%)	142 (25%
Completed high school	85 (31%)	57 (20%)	142 (25%)
*Body Mass Index (quartiles)*			
15.7-22.2	78 (28%)	65 (22%)	143 (25%)
22.3-24.1	76 (28%)	69 (23%)	145 (25%)
24.2-25.9	78 (28%)	76 (26%)	154 (27%)
26.0-36.5	43 (16%)	88 (30%)	131 (23%)
Years using at current residence (mean)	17.1	18.3	17.8
*Estimated monthly servings (mean)*			
Fruits and vegetables	16.7	14.3	15.4
Meat	20.3	21.8	21.1
(beef, pork, poultry)			
Other protein	15.8	12.6	14.1
(beans, milk, eggs)			

^a^Numbers do not add to 275 cases and 298 controls for some categories due to missing data. Column percents may not add to 100% due to rounding.

### Water arsenic

Water arsenic measures were available for a total of 560 subjects, 288 cases (51%) and 272 controls (49%). Most subjects were on piped water or municipal water (83%), and 17% relied on a well as their primary source of drinking water. Water arsenic exposures ranged from below detection (n = 2) to 208.1 μg/L. The overall median and mean water arsenic levels were 8.9 and 13.1 μg/L, respectively. Only 4 subjects had water arsenic measures above 100 μg/L (Table 2).

**Table 2:** **Case status and arsenic exposure categories**

**Water Arsenic (μg/L)**	**Control**	**Case**	**Toenail arsenic**	**Toenail arsenic**
			**(μg/g)**	**(μg/g)**
			**Mean/Median**	**Min/Max**
Under 10	137 (50.4%)	168 (58.3%)	0.77/0.57	0.18/16.4
11-39	131 (48.2%)	105 (44.5%)	0.94/0.59	0.13/34.2
40 -99	4 (1.5%)	11 (3.8%)	1.41/1.60	0.40/2.46
100 and over	0 (0%)	4 (2.2%)	3.38/342	2.72/3.95
**Toenail Arsenic (μg/g)**	**Control**	**Case**	**Water arsenic**	**Water arsenic**
			**(μg/L)**	**(μg/L)**
			**Mean/Median**	**Min/Max**
0.11-0.28	19 (8%)	32 (12%)	10.2/2.94	0.34/31.5
(<10^th^ percentile)				
0.29-1.37	212 (85%)	194 (74%)	11.8/9.00	0.29/88.3
(10^th^-90^th^ percentile)				
1.38-34.21	18 (1.5%)	35 (13%)	29.0/10.8	0.42/208
(>90^th^ percentile)				

Water arsenic was slightly higher among municipal water users (mean = 13.6 μg/L; median 9.9 μg/L) compared to well water users (mean = 10.8 μg/L; median 3.1 μg/L); although more well water users had water arsenic above 40 μg/L (6% vs 3% above 40 μg/L for well water and municipal water, respectively). Water arsenic was inversely associated with frequency of pork and beef consumption, and education. Never smokers and non-users of alcohol and unemployed subjects had slightly higher water arsenic exposures. No differences in water arsenic was evident by age, sex and family history of heart disease (Additional file 1: Table S1).

### Nail arsenic

Toenail arsenic measures were available or imputed from fingernail measures for a total of 533 subjects, 277 cases (52%) and 256 controls (48%). Toenail arsenic levels ranged from 0.18 μg/g to 34μg/g. Median and mean levels were 0.60 μg/g and 0.87μg/g, respectively. Log transformed water and nail arsenic measures were only slightly correlated (r = 0.12, p = 0.007). The correlation was stronger among subjects with water arsenic measures above 20 μg/L (n = 126, r = 0.45, p < 0.00005).

Males had slightly higher nail arsenic and current smokers had higher nail arsenic compared to former or never smokers (Additional file 1: Table S1). Nail arsenic also varied by occupation and in also contrast to water arsenic, professionals had the highest levels of nail arsenic and farmers had the lowest (Additional file 1: Table S1).

### Cardiovascular case status and water arsenic

Although few subjects had high levels of arsenic exposure, more cases compared to controls had water arsenic above 40 and 50 μg/L (Table 2). Fifteen of 19 subjects with water arsenic above 40 μg/L were cases and all four subjects with water arsenic above 100 μg/L were cases. Overall mean and median water arsenic measures were 13.2 and 7.5 μg/L among cases and 13.0 and 9.7 μg/L among controls.

Results from logistic regression models with water arsenic modeled as a continuous and categorical exposure are shown in Table 3. After adjustment for age, sex, smoking, diet, family risk factors and BMI, the adjusted odds ratio (AOR) for a 10 μg/L increase in water arsenic was 1.19 (95% CI: 1.03, 1.38). Other factors associated with case status included family risk factors for cardiovascular disease and history of smoking (Additional file 1: Table S2 and S3). Positive associations were also observed using log-10 transformed water arsenic (AOR =1.54, 95% CI: 1.09-2.17).

**Table 3:** **Adjusted odds ratios (AOR) for cardiovascular disease and water and nail arsenic exposure** ^**a**^

	**AOR**	**95% CI**	**p-value**	**p-value (trend)** ^**b**^	**N** ^**c**^
*Continuous exposures*					
Water arsenic (10 μg/L)	1.19	1.03-1.38	0.021	NA	553
Nail arsenic (μg/g )	1.16	0.98-1.38	0.09	NA	526
*Categorical exposures*					
Water arsenic (μg/L)					
<10	ref			0.06	
10-39	1.23	0.78-1.93	0.38		553
40 and over	4.05	1.10-14.99	0.036		
Nail arsenic (μg/g )				0.21	
0.11-0.28	Ref				526
(<10^th^ percentile)					
0.29-1.37	0.67	0.33-1.34	0.26		
(10^th^-90^th^ percentile)					
1.38-34.21					
(>90^th^ percentile)					
Ref: 0.11-0.28	1.91	0.73-4.99	0.19		
Ref : 0.11-1.37	2.48	1.18-5.20	0.0065		

^a^Covariates for multivariate models included diet, body mass index (BMI); occupation (unemployed, farming, industry, professional and other); education (None or some primary school; primary or some high school; completed high school); smoking (non-smoker, former smoker, or current smoker); and family history of hypertension, diabetes or heart disease. Full models are available in Additional file 1.

^b^p-value for trend across categories.

^c^Numbers do not add to 573 due to missing data on water arsenic (n = 13), nail arsenic (n = 40), or one more covariates.

Relative to water arsenic exposure less than 10 μg/L, there were elevated AORs for water arsenic exposures above 40 μg/L (AOR = 4.05, 95% CI = 1.1-14.99, p = 0.04, Table 3) and 50 μg/L (AOR 5.20 (p = 0.1; 95% CI 0.9-29.99).

### Cardiovascular case status and nail arsenic

Overall mean and median nail arsenic measures were 0.96 and 0.58 μg/g among cases and 0.79 and 0.60 μg/g among controls. A higher proportion of cases had nail arsenic above the 90^th^ percentile (1.38-34.2 μg/g) compared to controls (13% vs. 1.5%, Table 2). After adjusting for covariates there was a positive association between nail arsenic and case status (AOR for 1 μg/g increase in nail arsenic = 1.16, 95% CI 0.98-1.38, Table 3). In the categorical analysis, nail arsenic above the 90^th^ percentile compared to the 10^th^ percentile was positively associated with case status (AOR = 1.9, 95% CI 0.73-4.99, Table 3). When those above 90^th^ percentile are compared with all subjects below the 90^th^ percentile, the risk was stronger and statistically significant (AOR = 2.75; 95% CI 1.33-5.69, Table 3).

## Discussion

In recent years, health officials in Hangjin Hou, like other arsenic affected areas of Inner Mongolia, have focused on improving water quality and reducing arsenic exposures. In this study, water arsenic levels were lower than previously documented [4,28], and few subjects were exposed to over water over 100 μg/L. Despite these relatively moderate water arsenic exposures, we observed an association between well water arsenic and cardiovascular disease. The odds of having cardiovascular disease among those with drinking water arsenic above 40 μg/L was four times higher compared to those with drinking water less than 10 μg/L. Although we lacked sufficient data to evaluate departures from non-linearity and threshold effects below 40 μg/L, under the assumption of a linear trend, a 10 μg/L increase in water arsenic was associated with a 19% increase in the odds of cardiovascular disease.

Nail arsenic exposures were also lower than we observed previously in this region, however the median levels were approximately 10-fold higher than those observed in a study in the US [40]. Although we did not observe an effect of nail arsenic under 1.38 μg/g, subjects with the highest levels of nail arsenic had a greater incidence of cardiovascular disease. Water arsenic was more strongly associated cardiovascular disease in terms of the linear trend, which was not statistically significant for nail arsenic. We also observed only a slight correlation between nail arsenic and water arsenic (r = 0.17), although the correlation was considerably improved at higher water arsenic exposures above 20 μg/L (r = 0.45). The discrepancy at lower exposures may reflect recent switching of water sources, or exposures to other non-water sources of arsenic.

Results of this study are consistent with increasing evidence of associations between arsenic and cardiovascular disease at low to moderate levels of chronic arsenic exposure. In the United States, two recent studies have observed associations between heart disease and arsenic exposures under 100 μg/L. In south-central Colorado, a retrospective cohort study observed an association between time-weighted average lifetime exposure of arsenic and coronary heart disease in a region with drinking water arsenic under 100 μg/L [24]. Also, an analysis from the Strong Heart Study found an association with cardiovascular disease and urinary arsenic among American Indian communities in Arizona, Oklahoma and North and South Dakota [25]. Increases in blood markers of cardiovascular disease risk and inflammation have also been observed at low to moderate levels of exposure [4,40-42]. In Spain, arsenic at levels below 150 μg/L in municipal drinking water has been associated with cardiovascular mortality [43].

In this study, one limitation is that we lacked information on prior water exposures. While this likely introduced exposure misclassification, the impact of this bias may mitigated for several reasons. Subjects had been at their current residence a median of 20 years and a mean of 30 years and previous studies have described this region as a relatively stable population with low migration [4,44]. Also, well water arsenic levels in this area appear to be stable, and we previously reported samples from 57 wells collected 12 years apart collected from Shahai village in Hang-Hou were well correlated (r = 0.76, p < 0.0005) [28]. The latency period between arsenic exposure and the onset of cardiovascular disease is likely considerably less than that for cancer, which can be up to 20 or 30 years. In Chile, it was found that increased acute myocardial infarction mortality began concurrently with high arsenic exposures and were maintained for 10 years, compared to lung and bladder cancers which were not elevated until 20 years following the high exposures [8].

In this study we were able to account for many risk factors for cardiovascular disease, we individually and consistently diagnosed cases and controls, and individually measured arsenic exposure, thereby reducing misclassification. By using incident cases, a hospital based case-control design and restricting cases to residents of the Hang-Hou region, we were able to ensure that cases and controls were recruited from the same general population. Using incident cases rather than prevalent cases helped reduce the time between exposure assessment and the occurrence of cardiovascular disease though, as with most case-control studies, we cannot be certain that exposure preceded disease.

The study was limited in that few subjects were exposed to high levels of arsenic. We were able to partially control for the effects of age and sex by design through matching, but there were other strong risk factors associated with cardiovascular disease present in our data. Occupation, age, diet, family history and smoking history, were all associated with cardiovascular disease. Notably, those who quit smoking were at considerably higher increased risk for cardiovascular disease compared to non-smokers or current smokers, possibly reflecting those who quit smoking due to health concerns (Additional file 1: Tables S2-S5). It is possible that we were unable to fully control for these and other factors associated with arsenic exposure and cardiovascular disease. In addition, as a result of few subjects exposed to high arsenic levels, confidence bounds on our estimates of risk are wide, resulting in imprecision in our risk estimates.

## Conclusions

In a case-control study in Inner Mongolia, China, drinking water arsenic was associated with increased risk of cardiovascular disease among residents exposed to moderate levels of arsenic. The results of this study provide further evidence of the association between cardiovascular disease and arsenic at relatively moderate exposures.

## Additional file

Additional file 1:
**Table S1.** Arsenic exposure by demographic characteristic. **Table S2.** Adjusted odds ratios (AOR) for cardiovascular disease and water arsenic (continuous exposure model). **Table S3.** Adjusted odds ratios (AOR) for cardiovascular disease and water arsenic (categorical exposure model). **Table S4.** Adjusted odds ratios for cardiovascular disease and nail arsenic (continuous exposure model). **Table S5.** Adjusted odds ratios for cardiovascular disease and nail arsenic (categorical exposure model).
