# Supplementary material for: Cardiovascular disease and arsenic exposure in Inner Mongolia, China: a case control study
Source: Environ Health. 2015 Apr 12;14:35. doi: 10.1186/s12940-015-0022-y (PMC4409992; doi:10.1186/s12940-015-0022-y)
Supplement: Additional file 1: — Table S1. Arsenic exposure by demographic characteristic. Table S2. Adjusted odds ratios (AOR) for cardiovascular disease and water arsenic (continuous exposure model). Table S3. Adjusted odds ratios (AOR) for cardiovascular disease and water arsenic (categorical exposure model). Table S4. Adjusted odds ratios for cardiovascular disease and nail arsenic (continuous exposure model). Table S5. Adjusted odds ratios for cardiovascular disease and nail arsenic (categorical exposure model). [file 12940_2015_22_MOESM1_ESM.pdf]

Table S1: Arsenic exposure by demographic characteristics

	Water arsenic-mean (μg/L)	Water arsenic-median (μg/L)	Nail arsenic-mean (μg/g)	Nail arsenic-median (μg/g)
Sex				
Male	12.8	8.2	0.97	0.65
Female	13.8	9.5	0.68	0.53
Age				
21-40	11.6	10.8	2.1	0.87
41-50	15.7	10.8	0.81	0.64
51-60	13.2	8.4	0.83	0.56
61-70	12.1	4.5	0.73	0.56
Occupation				
Unemployed	21.9	31.5	0.87	0.52
Farming	10.9	4.3	0.75	0.57
Industry	10.1	9.2	0.85	0.75
Professional	12.6	10.8	1.11	0.59
Other	17.0	10.8	1.17	0.73
Smoking				
Never Smoker	14.5	10.8	0.70	0.54
Former Smoker	12.9	4.6	0.82	0.60
Current Smoker	11.8	6.8	1.1	0.68
Drinks Alcohol				
No	14.5	9.9	0.79	0.55
Yes	11.3	4.5	1.0	0.67
Family history of cardiovascular disease				
No	14.3	9.9	0.91	0.61
Yes	10.9	4.5	0.82	0.58
Education				
None or some primary school	11.6	4.4	0.73	0.54
Some high school	13.5	9.9	0.83	0.68
Completed high school	14.1	10.8		
Monthly frequency-eating fruit				
Less than once a month	15.9	9.9	0.95	0.64
1-5 times per month	11.6	6.5	0.75	0.56
>5 times per month	13.0	9.9	0.94	0.62
Monthly frequency-eating pork				
Less than once a month	21.3	10.6	0.74	0.47
1-5 times per month	14.4	10.5	0.74	0.47
>5 times per month	11.6	5.8	0.91	0.63

Table S1: Arsenic exposure by demographic characteristics (continued)

	Water arsenic-mean (μg/L)	Water arsenic-median (μg/L)	Nail arsenic-mean (μg/g)	Nail arsenic-median (μg/g)
Body Mass Index (quartiles)				
15.7-22.2	11.4	4.4	0.73	0.57
22.3-24.1	14.1	9.9	1.1	0.58
24.2-25.9	14.7	9.9	0.93	0.66
26.0-36.5	12.1	7.6	0.74	0.57

Table S2: Adjusted odds ratios (AOR) for cardiovascular disease and water arsenic (continuous exposure model)

	**AOR**	**95% CI**	**p-value**
Water arsenic (10 µg/L)	1.19	1.03-1.38	0.021
Age Category (ref=21-40)			
41-50	0.71	0.30-1.72	0.45
51-60	0.92	0.40-2.11	0.84
61-70	1.95	0.83-4.55	0.12
Sex (ref=female)	1.79	0.96-3.33	0.06
Smoking (ref=never smoker)			
Quit	8.37	3.97-17.65	<0.0005
Current	1.99	1.09-3.62	0.025
Farming occupation (ref=other occupations	1.94	1.23-3.07	0.004
Monthly servings of			
Meat	1.05	1.02-1.08	0.00084
Fruits and vegetables	0.92	0.88-0.97	0.0028
Other protein	0.95	0.92-0.97	0.00017
Family history of vascular disease	7.24	4.48-11.69	<0.0005
BMI	1.07	1.00-1.15	0.054

Table S3: Adjusted odds ratios (AOR) for cardiovascular disease and water arsenic (categorical exposure model)

	**AOR**	**95% CI**	**p-value**
Water arsenic (ref=under 10 μg/L)			
10-39	1.23	0.78-1.93	0.38
40 and over	4.05	1.10-14.99	0.036
Age Category (ref=21-40)			
41-50	0.76	0.31-1.82	0.53
51-60	0.99	0.43-2.26	0.97
61-70	2.02	0.87-3.37	0.10
Sex (ref=female)	1.81	0.97-3.37	0.064
Smoking (ref=never smoker			
Quit	8.06	3.82-17.00	<0.0001
Current	1.95	1.07-3.54	0.029
Farming occupation (ref=other occupations	1.78	1.11-2.88	0.018
Monthly servings of			
Meat	1.05	1.02-1.08	0.0009
Fruits and vegetables	0.92	0.88-0.97	0.00018
Other protein	0.95	0.92-0.97	0.00022
Family history of vascular disease	6.93	4.32-11.13	<0.0001
BMI	1.07	0.99-1.14	0.07

Table S4: Adjusted odds ratios for cardiovascular disease and nail arsenic (continuous exposure model)

	AOR	95% CI	p-value
Nail arsenic (µg/g)	1.16	0.98-1.38	0.09
Age Category (ref=21-40)			
41-50	1.01	0.39-2.63	0.98
51-60	1.21	0.49-3.01	0.68
61-70	2.72	1.07-6.94	0.036
Sex (ref=female)	1.85	0.96-3.58	0.068
Smoking (ref=never smoker			
Quit	8.06	3.69-17.60	<0.0001
Current	2.00	1.06-3.77	0.033
Farming occupation (ref=other occupations	1.95	1.22-3.10	0.005
Monthly servings of			
Meat	1.05	1.02-1.08	0.0013
Fruits and vegetables	0.92	0.87-0.97	0.0025
Other protein	0.94	0.92-0.97	0.00011
Family history of vascular disease	7.60	4.61-12.52	<0.0001
BMI	1.09	1.01-1.17	0.019

Table S5: Adjusted odds ratios for cardiovascular disease and nail arsenic
(categorical exposure model)

	**AOR**	**95% CI**	**p-value**
Nail arsenic (µg/g)			
0.11-0.28<br>(<10th percentile)	Ref		
0.29-1.37<br>(10th-90th percentile)	0.67	0.33-1.34	0.26
1.38-34.21<br>(>90th percentile)<br>Ref: 0.11-0.28 µg/g<br>Ref : 0.11-1.37	 <br>1.91<br>2.48	 <br>0.73-4.99<br>1.18-5.20	 <br>0.19<br>0.0065
Age Category (ref=21-40)			
41-50	1.00	0.38-2.62	0.99
51-60	1.20	0.50-3.40	0.69
61-70	2.67	1.11-8.11	0.04
Smoking<br>(ref=never smoker)			
Quit	6.97	3.10-15.65	<0.0005
Current	2.03	1.05-3.92	0.02
Farming occupation<br>(ref=other occupations	1.76	1.10-2.82	0.0086
Monthly servings of			
Meat	1.05	1.02-1.08	0.0013
Fruits and vegetables	0.92	0.87-0.97	0.0030
Other protein	0.94	0.92-0.97	0.00015
Family history of vascular disease	7.64	4.58-12.75	<0.0005
BMI	1.08	1.00-1.16	0.025
